# Supporting Decision Making in Early Years Initiatives: Co‐Designing a Child Health Evidence Dashboard

**DOI:** 10.1002/hpja.70230

**Published:** 2026-08-11

**Authors:** Brittany J. Johnson, Samantha Morgillo, Sarah Hunter, Lua Perimal‐Lewis, Georgia Middleton, Jacqueline Anderson, Rebecca Perry, Sarah Eley, Annapurna Nori, Louise Wightman, Sarah Taki, Carmel Williams, Vicki Brown, Anna Lene Seidler, Kylie E. Hunter, Rebecca Golley

**Affiliations:** ^1^ Flinders University, College of Health and Enablement Caring Futures Institute Bedford Park Australia; ^2^ Flinders University College of Science and Engineering Bedford Park Australia; ^3^ JBI Centre of Evidence‐Based Education & Arts Therapies Palacký University Olomouc Czech Republic; ^4^ Population Health Division Preventive Health SA Adelaide Australia; ^5^ Health Translation SA Adelaide Australia; ^6^ Parent Representative Adelaide Australia; ^7^ Strategy and Partnerships Division Preventive Health SA Adelaide Australia; ^8^ Maternal Child and Family Health Nurses Australia North Sydney Australia; ^9^ Health Promotion Unit, Population Health Research & Evaluation Hub Sydney Local Health District Sydney Australia; ^10^ The University of Sydney School of Public Health, Faculty of Medicine and Health The University of Sydney Sydney Australia; ^11^ Centre for Health in All Policies Research Translation School of Public Health, Adelaide University Adelaide Australia; ^12^ Deakin Health Economics, Institute for Health Transformation Deakin University Geelong Australia; ^13^ Universitätsmedizin Rostock, Mecklenburg‐Western Pomerania Rostock Germany; ^14^ Sydney Clinical Trials Centre University of Sydney Sydney Australia

**Keywords:** child health, evidence, health promotion, knowledge translation, next‐users, research to practice gap

## Abstract

**Issue Addressed:**

Research has trialled many initiatives to support caregivers to shape young children's health behaviours. However, translating evidence to public health practice and policy is a challenge, in part due to access to such evidence. We aimed to co‐design a child health evidence dashboard, and to understand the expected enablers and barriers to use of the dashboard with next‐users (e.g., Australian child service managers, program planners).

**Methods:**

Using an integrated knowledge translation approach, we conducted a mixed‐methods study involving five online co‐design workshops. Next‐users were invited to participate in the workshops and completed pre‐ and post‐workshop surveys. Pragmatic data analysis occurred after each workshop to identify next‐user requirements and inform iterative dashboard development alongside workshops.

**Results:**

15 next‐users participated in the workshop series (median of 9 per workshop). Next‐user preferences were categorised into desired uses, content, features and functionality, and design for the dashboard. Key enablers to use of the dashboard included relevance, promotion and credibility of the dashboard, positive user experience, time and resources to support use of the dashboard.

**Conclusions:**

We identified what and how next‐users want evidence on child health initiatives to inform policy and practice. Findings enabled development of an initial evidence dashboard prototype informed by next‐user preferences. Understanding the enablers and barriers to using and engaging with the dashboard will inform ongoing development and implementation.

## 1 Introduction

Child health behaviours relating to diet and movement (including sleep) support growth and prevent chronic conditions including obesity [1]. Young children in Australia and globally are not meeting recommendations for health behaviours. Two‐thirds of Australian toddlers meet recommendations for vegetable intake [2]. Similarly, a study of young children from 33 countries found 49% met physical activity recommendations, 42% met screen time recommendations and 81% met sleep recommendations [3]. The early years are when children learn behaviours that track into later life [4], highlighting a key time for initiatives (i.e., programs and services) to establish health promoting behaviours [5]. Supporting health behaviours, growth and the primary prevention of obesity are recognised as priority action areas in Australia, for example the National Obesity Strategy [6]. Such national strategies have informed ongoing efforts to embed a health promotion focus into relevant policy at a state and territory level [7, 8]. Whereby resourcing towards initiatives aimed at promoting child health behaviours in the early years, can support improving public health outcomes.

There is extensive evidence identifying initiatives to support parents/caregivers in shaping children's health behaviours [9, 10]. However, it has had limited impact on the initiatives delivered in real‐world settings, highlighting an evidence to policy and practice gap [11]. Barriers to translating evidence include its applicability or relevance to policy contexts, access to academic journals, and challenges in initiative implementation [12, 13, 14]. Addressing these barriers to translation is critical for increasing adoption of evidence in real‐world settings and improving health outcomes in the early years.

TOPCHILD (Transforming Obesity Prevention for CHILDren) is the largest global collaboration of trials evaluating early obesity prevention initiatives that aim to change parental behaviours related to feeding and diet, activity and sleep practices in the first 1000 days of life (conception to 2 years) [9]. It is a living evidence database that provides a detailed description of initiative content [13, 15] and their effectiveness [9, 10]. Database uses include evidence synthesis (e.g., meta‐analyses) to identify effective initiatives and identifying commonality of content across initiatives. This can aid program implementation in policy and practice settings, and also initiative components to embed into new or existing service [16]. The implementation of evidence‐based initiatives into policy and practice is becoming more common in Australia (e.g., scale up of INFANT in Victoria [17] and Munch and Move in New South Wales [18]), but is associated with feasibility and resourcing challenges [19] that can lead to contextual initiative adaptions [20, 21]. This highlights the need for implementation strategies to overcome some of the barriers to translating research into practice.

Common elements are discrete techniques or practices that are commonly found across evidence‐based initiatives and can be used to guide contextual initiatives delivery [16]. Although this approach is less common than use of ‘off‐the‐shelf’ initiatives, examples of its use to inform implementation include the South Australian Child and Family Support System [22] and early childhood education in the United Kingdom [23]. These examples demonstrate use of this approach to allow pragmatic and feasible implementation, supporting the mobilisation of evidence using flexible approaches that can be applied to the health promotion field.

The TOPCHILD‐Policy [24] broader program of work aims to make the TOPCHILD Collaboration living evidence database accessible and useful in guiding initiatives by policy makers, service managers and health promotion program planners to support caregivers of young children to promote health behaviours. The TOPCHILD‐Policy program also aims to understand interest‐holders' needs (i.e., next‐users of the evidence) to understand their decision‐making process [25] and develop practical tools to support evidence uptake and adoption. This can also include generating additional evidence (e.g., caregiver preferences for common elements [26] and initiatives outcomes [27]).

Prior work informed this study, where interviews were conducted with individuals (e.g., child health program planners, policy makers) who make decisions to implement or endorse initiatives in the early years system (i.e., next users of the evidence) to understand their current decision‐making process and needs for an evidence database [25]. Findings supported the need for practical tools to support decision‐making, with participants describing a range of approaches to sourcing information but noted the process was not exhaustive. Participants described practical tools including a digital platform or online repository that brings together information sources, something that's easy to use and accessible [25], hence the desire for a dashboard (user‐friendly, interactive interface that brings together evidence to support decision‐making, monitoring and translation) [25]. This is reinforced by a recent study of public health trials finding research products and tools for evidence dissemination as a key influence in achieving research impact. Therefore, the aims of this study were to (1) co‐design a child health evidence dashboard (hereafter referred to as ‘dashboard’), and (2) understand the expected enablers and barriers to use the dashboard.

## 2 Methods

### 2.1 Study Design and Theoretical Guidance

The study used co‐design as part of the broader TOPCHILD‐Policy program of work to develop and evaluate a dashboard to increase evidence‐informed decision‐making in initiatives that support child health behaviours in the first 1000 days [24]. Underpinned by the integrated knowledge translation approach [28], this program of work engages knowledge users to co‐produce usable outputs, and is guided by input from an Advisory Panel (including caregivers, practitioners, influencer/advocates, managers, boundary spanners). Drawing on the knowledge creation component of the Knowledge to Action framework [29], this program is developing tailored products/tools (i.e., dashboard) by adapting and contextualising evidence. The co‐design workshops were informed by human‐centred design principles [30] to develop the dashboard. Table 1 presents the application of each component of the theoretical guidance. This study was approved by the Flinders Human Research Ethics Committee (HREC 7307). Online informed consent was obtained by all participants. Reporting follows the Guidance for Reporting Involvement of Patients and the Public (GRIPP2‐LF) (File S1) [31].

**TABLE 1:** Application of theoretical guidance in the study.

Guidance	Component	Application
Knowledge to action framework [29]	Knowledge creation component includes phases of knowledge inquiry, synthesis and products/tools, and tailoring knowledge	The study aim was to create knowledge on ‘products/tools’
		Focus of the workshops related to understanding next user needs to tailor knowledge applied across phases in the knowledge creation component
	Action cycle component includes phases relating to identifying problem/knowledge, adaptation to local context, assessing barriers, selecting implementation interventions, monitoring use, evaluating outcomes and sustaining use.	
		Workshop 5 included a focus on assessing barriers/enablers from the action cycle
Human‐centred design principles [30]	Mindsets	Mindsets were encouraged to be adopted at the start of each workshop
	Methods—Inspiration, ideation, implementation	Methods were used to design of activities/tasks within the workshop series

### 2.2 Participants and Recruitment

An interest‐holder mapping activity defined different types of interest‐holders within the early years system (e.g., recipients, advocates, planners, researchers), this mapping informed purposive sampling of anticipated next‐users (a subset of interest‐holders identified). Next‐users were defined as individuals with roles in the Australian early years system likely to use or endorse the dashboard including those in government and non‐government health promotion agencies and organisations. Key roles included program planners, policy makers, service managers, organisation managers, and boundary spanners (defined as working across organisations/sectors). Recruitment involved emailing the list of identified next‐users, sharing an infographic on LinkedIn, public presentations to early childhood interest‐holders (e.g., Healthy Development Adelaide session), and collaborating with project partners to identify and contact key organisations for partners to approach with the recruitment invitation for certain next‐user roles.

Participants were invited to the series of five workshops and encouraged to attend all workshops when possible. The target recruitment sample size was 20–30 participants, aiming for approximately 10–15 participants per workshop. Participants were offered AUD$25 per workshop as reimbursement of their time and contribution, with some participants contributing to the workshops in their organisational capacity as in‐kind support.

### 2.3 Workshops

**FIGURE 1:**
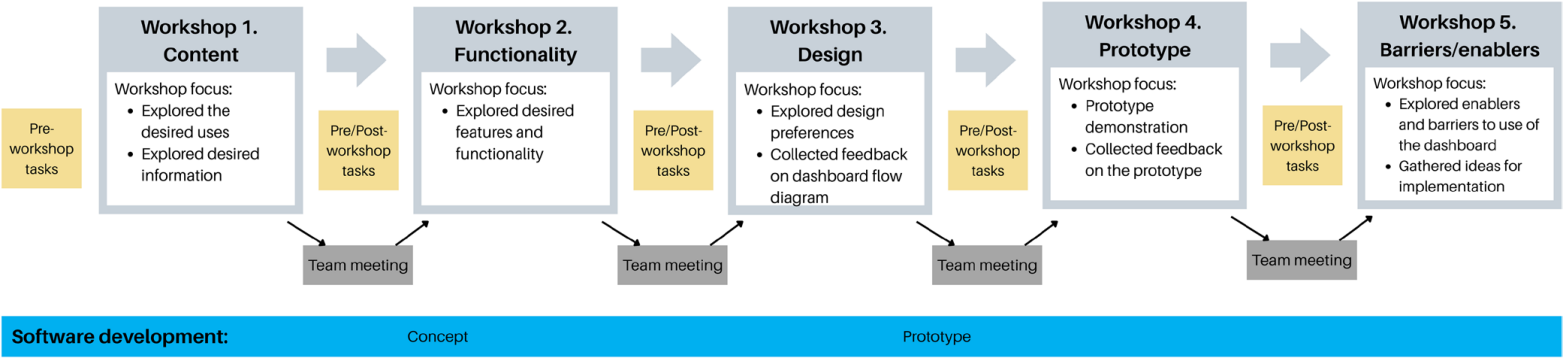
Co‐design workshop series alongside iterative dashboard development.

A series of five online co‐design workshops (Figure 1) were conducted using Microsoft Teams (V7.8.2), spaced approximately 4 weeks apart between August to December 2024. Each workshop went for 60 min, with the first workshop extended to 90 min to accommodate orientation to the project. Workshops were facilitated by a knowledge translation researcher (SH) with co‐design and facilitation expertise and co‐facilitated by project team members (SM, BJJ), including a content expert in child health research (BJJ). A software developer was invited to all workshops to support understanding of next‐user requirements to guide conceptual design and provide technical advice on functionality (e.g., examples of technical functions possible in a dashboard).

Human‐centred design principles [30] were applied to co‐design and iteratively develop the dashboard concept and prototype based on next‐user requirements and feedback. Design mindsets were presented and applied to create a safe space for participation, sharing ideas and embracing the iterative process [30]. Each workshop's structure included an introduction (icebreakers, recaps of previous workshops, workshop objectives), demonstration (featuring example websites, draft navigation flow diagram, prototype), activity (idea generation, feedback, discussion) and wrap up (including next steps and reflection). PowerPoint (Version 2502) slide decks were used to guide workshops. Various activities and online resources were used to collect and prioritise ideas or collect feedback to inform dashboard design (Table S1). Activities included brainstorming, sorting, prioritising and demonstrations with feedback. Online resources included Microsoft Teams break out rooms for smaller group activities and discussions, Miro interactive whiteboards [32] for group activities (Figures S1–S3) and Sli.do polls [33] to capture individual and anonymous responses. Reflection surveys at the end of each workshop iteratively informed the co‐design process by collecting what went well, what did not go well, and what could be improved.

Pre‐workshop tasks prepared participants for each topic and included pre‐readings, reviewing example websites with accompanying prompts or questions, and surveys to collect information and streamline discussions in workshops. Post workshop tasks included surveys to prioritise ideas from the workshops and provide additional thoughts. Participants unable to attend workshops were offered the opportunity to provide input by completing tasks out of session.

Registered participants were sent objectives and pre‐workshop tasks via email 1–2 weeks prior to each workshop. All participants were emailed the post‐workshop summary and, where relevant, post‐workshop task. All communications and documents were accessible on the shared Microsoft Teams page. Project team meetings were held between workshops to review findings, make pragmatic decisions regarding the concept and prototype, and prioritise activities for the following workshop.

### 2.4 Data Collection

Qualitative and quantitative data were collected throughout the workshop series. Workshops were recorded and transcribed by Microsoft Teams (AI transcription). Qualitative data captured next‐user requirements to create a dashboard ‘wish list’ that informed prototype development. Qualitative data sources included workshop transcripts, chat function transcripts, images from online whiteboard activities, Sli.do outputs and open text survey responses. Quantitative data were used to understand the study sample and inform priorities for the dashboard requirements. Quantitative data included participant demographics, Microsoft Teams chat voting results and closed survey responses.

Demographic data collected using a REDCap survey [34] included participant age, gender, jurisdiction, workplace sector, name of organisation and role title (to categorise organisation type), length of time employed by current organisation, and use of evidence or initiatives involvement in role. Surveys during the workshop series were hosted on Qualtrics [35] and collected the following: (1) Prioritisation of content, (2) Prioritisation of features and functions, (3) Website design preferences, (4) Prototype feedback and (5) Dashboard enablers, barriers and implementation.

### 2.5 Data Analysis

Aligned with the co‐design approach, pragmatic data analysis [36] occurred after each workshop to identify next‐user requirements and inform iterative dashboard development. This pragmatic data analysis approach meant rapid analysis of qualitative data using a combination of inductive and deductive approaches to meet the timelines between workshops, with analysis refined at the completion of all workshops with all data. Quantitative data were analysed using frequency (counts) or mean (SD) as appropriate. Workshop transcripts were reviewed and checked against the recording for accuracy (SM). Nvivo software (Version 14) [37] was used to support qualitative data analysis. A qualitative descriptive approach [38, 39] was used to analyse the data collection from workshops and surveys (i.e., transcripts, teams chats, whiteboards, Miro outputs and pre‐workshop surveys where applicable), guided by a deductive coding scheme derived from workshop topics and our prior interviews with next‐users [25]. One researcher (SM) conducted the analysis, guided by an experienced qualitative researcher (GM). Initial codes were identified immediately post workshops, capturing ideas from all available workshop resources. Categories were created according to the workshop topic(s) and activities (i.e., desired uses, content, functions, design, website navigation feedback, prototype feedback, enablers, barriers and implementation). Sub‐categories existed only for content, which were pre‐determined by the project team according to the data in the TOPCHILD evidence database. Data unrelated to a workshop topic were assigned other categories (e.g., data about design from the functionality workshop). These codes were then discussed with the project team (SM, BJJ, SH) and used to generate the workshop summaries which were sense checked with participants. Initial results were prioritised (i.e., content and functions) via post workshop surveys. A reflexive log was kept for the analysis process.

Following the collection of data from all workshops and via individual feedback, data were reviewed and re‐organised to reflect next‐user requirements (e.g., prototype feedback referring to desired design preferences). Reorganisation also entailed grouping similar codes into sub‐categories. The nature of the data meant that some codes were applied to multiple categories (e.g., desired design and enablers). Multiple team meetings (SM, BJJ, GM, SH, RG) were held to discuss the data and coding structure prior to finalisation with the broader team.

## 3 Results

### 3.1 Sample Characteristics

There were 15 participants in the workshop series, with a median attendance of nine (range 8–10) participants per workshop (Table 2). There were five pre/post workshops surveys with a median of six (range 0–8) participants completing the surveys.

**TABLE 2:** Co‐design sample characteristics.

Characteristic		Participants (*n* = 15)
Age^a^ (years)	Average ± SD	39.6 ± 7.8
Gender	Female/Women	15
Jurisdiction	South Australia	7
	New South Wales	3
	Queensland	3
	Victoria	2
Workplace sector	Health	10
	Community/social services	3
	Education	2
Workplace type	Government	11
	Not for profit organisation	3
	University	1
Length of time in organisation (years)	< 2 years	6
	3–10 years	8
	> 10 years	1
Role involves use of evidence	Identify/search for an evidence‐based initiatives to implement	14
	Adapt an evidence‐based initiative	12
	Review/revise current initiative	12
	Design a new initiative	13
	Evaluate an initiative	2

^a^ *n* = 5 missing.

### 3.2 Next‐User Requirements for the Child Health Behaviour Evidence Dashboard

**FIGURE 2:**
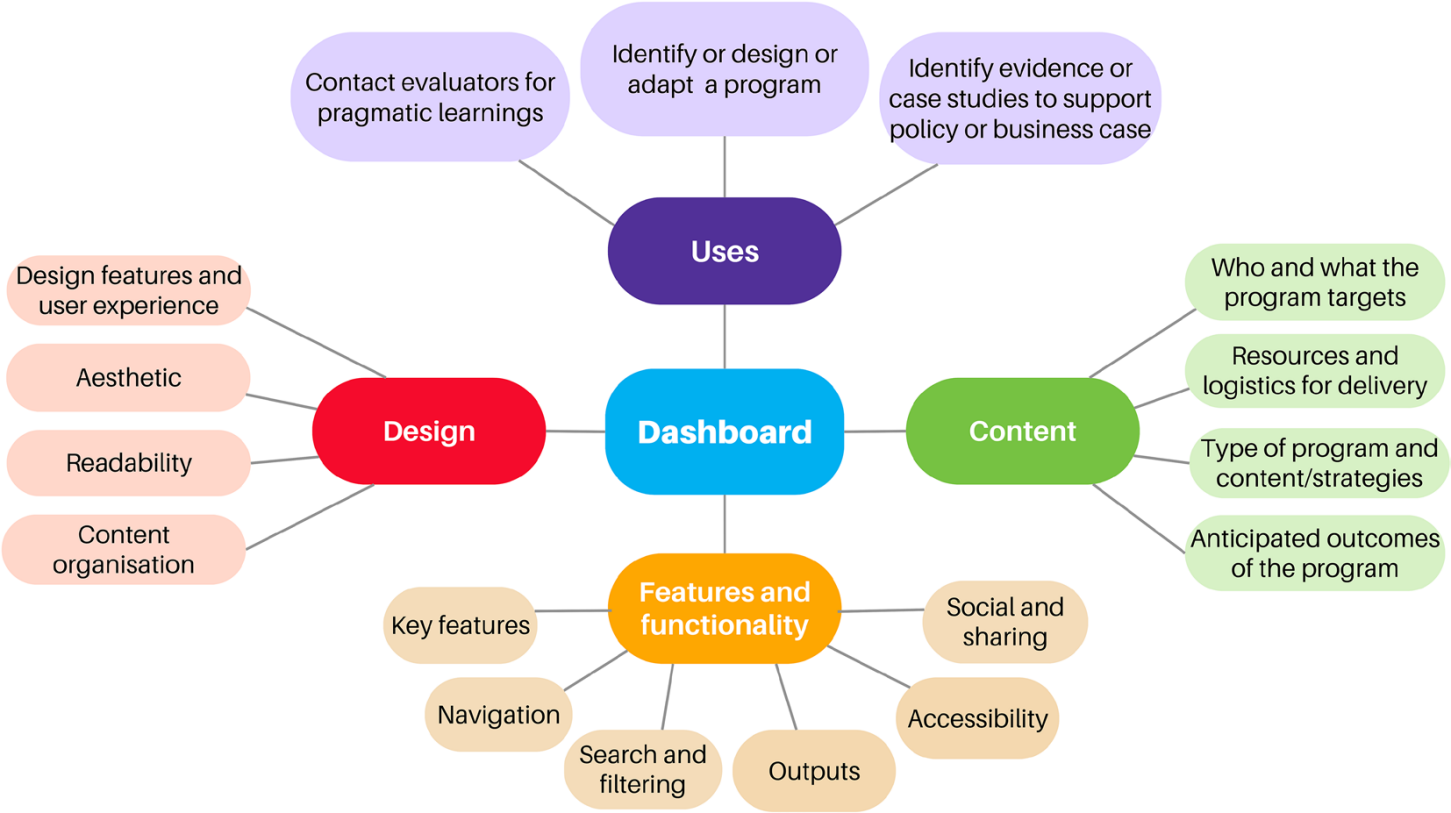
Desired next‐user requirements for a child health evidence dashboard.

Figure 2 summarises the next‐user preferences for a child health behaviour evidence dashboard. These were categorised under dashboard uses, content, features and functionality, and design.

#### 3.2.1 Dashboard Uses

Participants (*n* = 12) voted for likely uses of the dashboard, pre‐specified by the research team informed by prior interviews [25]; identify an initiative (*n* = 10), design an initiative (*n* = 10), adapt an initiative (*n* = 10), review or revise current initiative (*n* = 7). Additional uses described by participants were to identify evidence to support policy positions, identify case studies to pitch a business case, and contact initiative evaluators for pragmatic learnings.

#### 3.2.2 Desired Dashboard Content

Participants generated ideas for information they would like to access from the dashboard, building on the existing TOPCHILD Collaboration evidence database [15]. Activities during and post workshop prioritised information that participants would need to know about child health behaviour initiatives (Tables S2 and S3). The desired content for the dashboard was subcategorised into who and what the initiative targets, resources and logistics to deliver the initiative, type of initiative and content/strategies and anticipated outcomes of the initiative.

When considering who and what the initiative targets, participants prioritised needing to know the population targeted, the relevant life course stage (i.e., pregnancy, age of child) and priority population targeted (e.g., First Nations, culturally and linguistically diverse, experiencing disadvantage through geographical location or income, family members with disability) relevant to the participants specific organisational contexts. Participants wanted information on initiatives focussing on ‘child behaviours’ and addressing ‘child development’ (i.e., brain development and milestones). For example, ‘child development is probably the other one that plays into feeding and physical activity, et cetera’ (Participant 13, Workshop 1).

Resources and logistics to deliver the initiative were identified as important for implementation, with participants prioritising information on how the initiative is delivered (i.e., online, in‐person, group or individual), setting and organisation where the initiative is delivered, duration, and required equipment and technology. For example, ‘The technologies required, we know we're, you know, pushing the boundaries of technology these days and digital health interventions and things’ (Participant 13, Workshop 1). They highlighted needing resources for initiative implementation, and consideration of initiative sustainability and scalability such as training for facilitators, ‘… people that are delivering those programs… the training that you might need to provide those people that are running’ (Participant 6, Workshop 1).

For information about the type of initiative and content/strategies, participants prioritised the type of initiative delivery (i.e., education, support, activities), *‘*Type of program delivery is… must know because you need to know what how it's going to be delivered’ (Participant 5, Workshop 1). They also prioritised information on initiative background and rationale, and materials provided to parents. Participants highlighted the importance of initiatives that were community led and/or developed with consumer experience, especially in the context of priority populations, ‘…*really wanting to know, you know, their involvement in terms of that end user involvement and how, how and with whom those interventions might have been designed*’ (Participant 4, Workshop 1).

Participants prioritised anticipated outcomes relating to child health behaviours, parent and family wellbeing, sustainability of the initiative. For example, ‘Evidence that there is actually a return in investment for implementing the program (i.e., that it is actually feasible and effective in the real‐world setting similar to my work setting) and that it could realistically be implemented’ (Participant 7, Workshop 1 survey).

#### 3.2.3 Desired Dashboard Features and Functionality

The subcategories generated in desired dashboard features and functionality were: key features, search and filtering, navigation, outputs, social sharing functions and accessibility (Tables S2 and S4).

For key dashboard features, participants prioritised an evidence hub of initiatives with consolidated initiatives summaries, for example ‘Evidence to show the program works. A hub for evaluations and published results’ (Anonymous, Workshop 2 Sli.do activity). They expressed interest in being able to access case studies, for example ‘whether there's examples of the program being run elsewhere which might offer like support or examples of people running or involved in the program, if that makes sense, like kind of like a case studies type example maybe’ (Participant 1, Workshop 1). They additionally expressed interest in tools and resources to support implementation, as well as measurement tools for evaluating initiatives*—*‘specific measurement tools or instruments … would be useful’ (Participant 4, Workshop 2). An orientation video and an ‘about us’ page were recommended, to inform users about the dashboard's purpose and development.

Participants shared how they would like to interact with the dashboard, describing the importance of search and filtering functions to tailor content to their needs and streamline access to relevant evidence, *‘*I think like having really good filters is probably one of the key things’ (Participant 13, Workshop 2). They emphasised the need to filter for population, outcomes, location and topics and highlighted the importance of intuitive navigation, ‘I like a top bar on the home page to have another navigation feature’ (Participant 3, Workshop 3).

Participants desired outputs of free and easy downloads with access to initiatives and evidence summaries, publications, fact sheets and resources, such as ‘able to easily download content as PDFs or Excel where appropriate’ (Anonymous, Pre‐workshop 3 survey). In addition, they requested the inclusion of social and sharing functions, including the ability to contact others who have implemented initiatives and compatibility with various devices to enhance dashboard accessibility.

#### 3.2.4 Desired Dashboard Design

Desired design consisted of subcategories of design features and user experience, aesthetic, readability and content organisation and categorisation (Table S2).

Participants recommended a simple design and layout, intuitive to use and easy to navigate. As one participant described, ‘And just to have something like this, that's really simple and intuitive to use for me to go into and say ohh there's this program that work like really does show that it works on this. That's gonna be amazing’ (Participant 9, Workshop 1). For readability, participants preferred concise text and use of bullet points, text broken up with visuals, and use of intuitive language. Regarding the overall aesthetic, participants highlighted the dashboard should be visually attractive through use of relevant visuals, images, icons, photos and colour. Overall, participants described the need for intuitive content organisation and categorisation to support usability.

### 3.3 Considerations for Dashboard Development and Implementation

Participants identified factors that could affect their use and engagement of the dashboard as both enablers and barriers to use. They also generated ideas for dashboard evaluation and implementation to inform the next development and testing stages (Text S1).

#### 3.3.1 Enablers of Using the Dashboard

Identified enablers were relevance, credibility, dashboard, resources to support dashboard use, user experience, and time.

Relevance is an enabler that includes having a reason to use the dashboard, access to useful information and a range of resources, ‘It needs to be current/relevant and include enough information and detail to then be useful.*’* (Anonymous survey, Pre‐workshop 5). Credibility is associated with up‐to‐date and high‐quality information, as well as the dashboard having been developed by academics, ‘… *So you can actually see the team of academics behind the dashboard just to add that credibility…*’ (Participant 7, Workshop 5). They suggested regular communication reminders to aid dashboard awareness, such as email prompts for new information, ‘… *reminders so you are prompted to have a look at the dashboard on a semi regular basis…*’ (Participant 14, Workshop 5). In addition, resources such as training or webinars to support use of the dashboard, ‘Some sort of training for the people that could use that would be beneficial. Potentially you know webinars that you know that could you know they could engage in or like, you know, yeah, organizations can engage in and then support.’ (Participant 6, Workshop 5). Additionally, recommending positive user experience strategies to increase use, for example minimal log in procedures, no errors, easy to navigate, user‐friendly and simple and intuitive to use: ‘Simple, easy to use and understand’ (Participant 3, Post‐workshop survey 2). Lastly, availability of time to use the dashboard was an enabler.

#### 3.3.2 Barriers to Using the Dashboard

Identified barrier subcategories following dashboard use were identified: lack of relevance, access barriers, user experience barriers, and design and readability barriers (Supplementary file 3: Text S1). Most barriers were the inverse of enablers.

### 3.4 Iterative Dashboard Software Development

**FIGURE 3:**
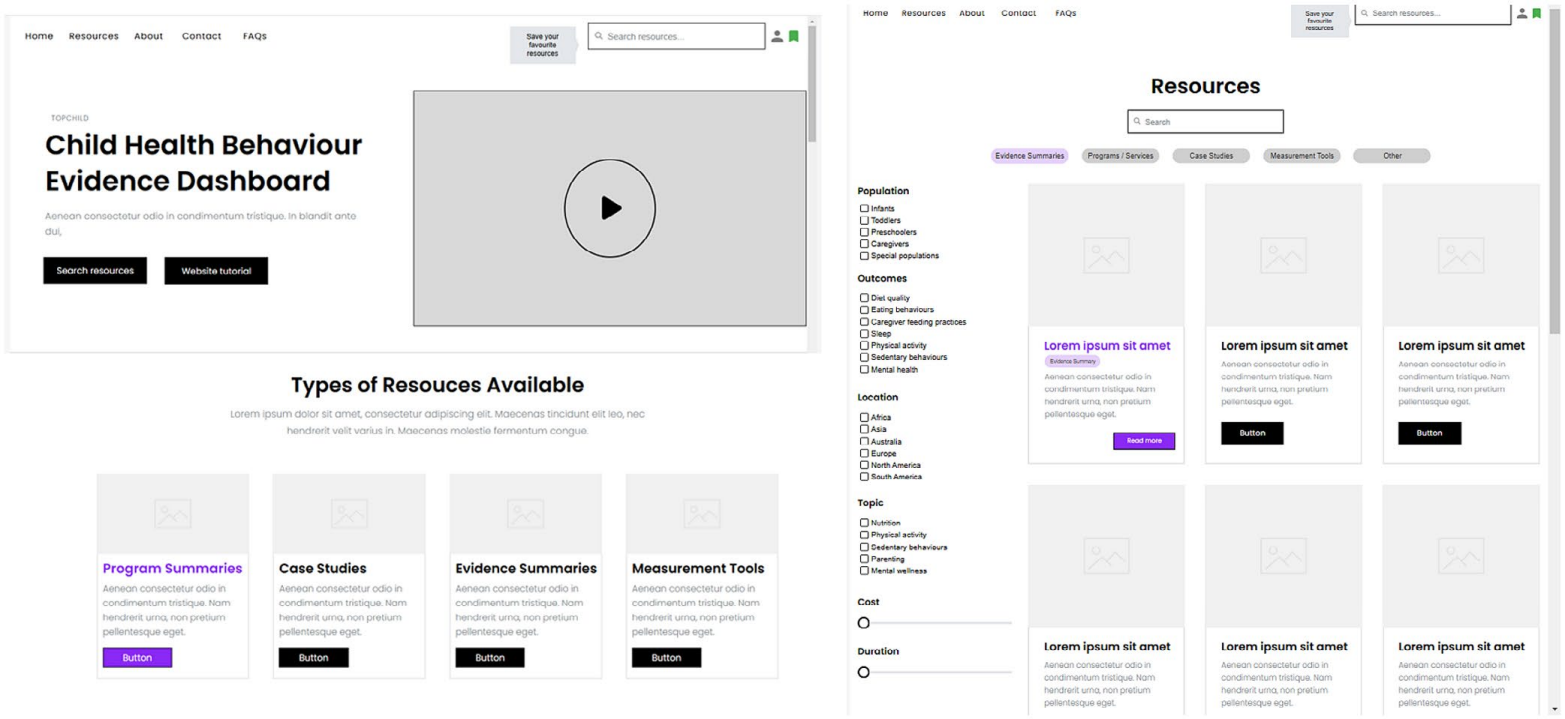
Prototype screenshots demonstrated at the workshops for participant feedback.

Iterative development of dashboard concept and prototype occurred alongside workshops. A high‐fidelity prototype was developed by the software developer, hosted on JustInMind design and prototyping tool [40]. The prototype focussed on demonstrating the dashboard front‐end, and used placeholders for text (Lorem ipsum), content and functionality that were still in development. The key requirements from the first three workshops (i.e., uses/content, functionality, design) were incorporated into the prototype (Figure 3), consisting of a home page, ‘about the dashboard and team’ page, resources page (repository of all available resources including initiatives, case studies, tools) with search and filter capability, and an initiative summary page with contact details and downloadable resources. Feedback provided by participants during the workshops confirmed that the prototype dashboard was meeting expectations in the early development phase. Positive feedback related to the design and layout, ease of navigation, use of white space, font size, and the filter functions. They also provided suggestions to improve navigation, functionality and design for the next iteration of the prototype (Table S5).

## 4 Discussion

Translating evidence to public health and health promotion practice and policy is a common challenge. We aimed to co‐design a child health behaviour evidence database dashboard with next users (e.g., program planners, service managers, policy makers), and to understand the enablers and barriers to use of the dashboard. Through co‐design workshops, we identified what and how next‐users want evidence on child health initiatives. Findings enabled development of an initial evidence dashboard prototype informed by their preferences categorised into desired uses, content, features and functionality, and design. Many of these findings can apply to other content areas in the health promotion field. Understanding the enablers and barriers to dashboard use and engagement will inform ongoing development. Key enablers to dashboard use include its relevance, promotion, and credibility, positive user experience, and time and resources to use it. The tailored dashboard will provide next‐users access to evidence needed to inform health promotion policy and practice decision.

Next‐users have previously reported the need for practical resources to access and use evidence in policy and practice decision making to support child health behaviours [25]. The findings from the current workshops confirmed that participants would access a dashboard that supports the identification, design and implementation of initiatives that promote child health behaviours in the early years. The most preferred content related to information identifying evidence relevant to their specific context. In many cases, the content could help next‐users determine the feasibility, sustainability and value/return on investment. Our findings largely align with the top six ranked outcomes in a public health policy decision survey of Australian policy makers and practitioners [41]. They found preference for initiative effectiveness, equity, feasibility, sustainability, acceptability and economic outcomes [41]. This is somewhat unsurprising given frameworks such as the GRADE Evidence to Decision frameworks also highlight similar content including evidence and considerations for benefits and harms, values and balance of effects, resources required, cost‐effectiveness, equity, acceptability, feasibility [42]. While the specific initiatives to suit participants' contextual needs differed, these key content needs were common across participants, hence were prioritised content to include in the prototype. In addition, participants reported dashboard functions that would enable easier access to such content (e.g., filtering a repository). The perceived enablers and barriers to use of the dashboard are similar to findings from broader use of digital technologies, where enablers include perceived usefulness, availability of training, adherence promotion and ease of use [43]. Consideration of technical barriers were identified as limiting use [43]. Accommodating next‐users' requirements in the development of a dashboard is essential to enable evidence use and thereby provide usable context‐specific outputs to support decision making and adoption.

An integrated knowledge translation approach [28] enabled next‐users to share their information needs and express preferences for the evidence dashboard prior to development. There is a critical gap in the evidence policy/practice translation process where the people implementing child health initiatives (i.e., next‐users) are often not included in the initial design and development of initiatives. Many child health behaviour co‐design approaches engage consumers/beneficiaries or practitioners (e.g., [44, 45]), with few examples of co‐design with program planners, service managers and boundary spanners as next‐users. Our findings aligned with the Knowledge to Action framework [29], by not only capturing preferences for dashboard prototype development (i.e., practical tools), but also next‐users highlighted the need for information about resourcing/costing information, this evidence on costing is not yet synthesised, hence has informed additional ‘knowledge synthesis’ needed to input into the dashboard. A study by Wolfenden and colleagues [46] examining research impact from public health trials found greater use of Knowledge to Action framework components was associated with a 30% greater odds of trial impact (total KT strategy domain OR = 1.30, 95% CI: 1.02, 1.66; *p* = 0.031). This suggests use of the Knowledge to Action framework in the design of the dashboard may increase the odds of impact when the dashboard is used in policy and practice decision making. Further, findings in the study by Wolfenden and colleagues [46] suggests use of research products and tools for evidence dissemination, such as our dashboard, may be one of the two most influential components to achieving research impact. During the final workshop, participants also generated ideas for how to implement and evaluate the dashboard once programmed, hence informing the next steps for the TOPCHILD‐Policy program. This ‘involvement of next‐users’ component of the Knowledge to Action framework was one of the less frequently used (i.e., untapped potential) in the previously mentioned study of impact from public health trials [46]. Findings from the workshops regarding next‐user preferences for the dashboard uses, content (except for the specific topics and outcomes), features and functionality, and design could be applied in other health promotion areas in the early years and broader policy and practice within scope of next‐users. This could include the dashboard being expanded with additional content integrated into the one dashboard or through a series of complementary dashboards to support policy and practice decision‐making.

There were several strengths to our co‐design process including involving a software developer in workshops to hear directly from next‐users and answer technical questions as they arose. Engaging next‐users early in the development process with mixed methods to capture ideas/perspectives, as well as opportunities within and outside of the workshops, allowed all participants to contribute meaningfully regardless of their different adult learning styles or personalities (i.e., group vs. individual feedback, written vs. verbal). In addition, short reflections provided at the end of each workshop allowed participants to be involved in how the co‐design process progressed. Progressive findings were sense‐checked by emailing all participants with each workshop findings, as well as sharing these at the next workshop. Member checking indicated increased confidence in the analyses [47].

Despite efforts for purposeful, stratified recruitment we had fewer participants than initially planned, resulting in limited diversity across jurisdictions with participants primarily from the health sector in four states. The intentional decision to keep the duration of workshops short (~60mins) to maintain engagement and minimise burden may have limited depth of captured preferences. However, the co‐design workshops are only the initial dashboard development stage, with ongoing iterations to be generated through future user testing and pilot testing with next‐users. Following user testing, the dashboard will be tested via pilot trials in real world contexts, with continuous iterations and refinements as appropriate. It is essential that future research with the dashboard ensures a process is established to update the dashboard with the most recent information and resources to remain useful.

## 5 Conclusion

Dissemination tools that facilitate access to research and enable adaptation to specific contexts are necessary to increase adoption of child health evidence for public health practice and policy to support promoting child health behaviours. Findings show that next‐users in the early years system want a child health behaviours evidence dashboard to inform decision‐making in identifying, designing and adapting initiatives. Ensuring that dashboard content and functionality meet next‐users' needs is important to enable use in practice. The desired dashboard content and functionality seeks to support selection and design of future initiatives tailored to next‐users' context. The tailored dashboard, ‘Evidence Hub’ will provide next‐users access to evidence needed to inform health promotion policy and practice decision. Further investigation of user‐testing prior to pilot trials in practice settings will evaluate the implementation of the dashboard and adoption of evidence into practice and policy decision‐making.

## Funding

TOPCHILD‐Policy is supported by the Ian Potter Foundation Public Health Grant (no. 21894) with in‐kind support from project partners: Preventive Health SA, Health and Wellbeing Queensland, NSW Health Sydney Local Health District and Ministry of Health Centre for Population Health, Health Translation SA. The TOPCHILD Collaboration evidence database development was funded by an Australian National Health and Medical Research Council Ideas Grant (2020–2023; GNT1186363) and Australian National Health and Medical Research Council Centre for Research Excellence in Translating Early Prevention of Obesity in Childhood (2022–2026; GNT2006999). BJJ is supported by The Hospital Research Foundation Group Early‐Mid Career Fellowship. ALS was supported by an Australian National Health and Medical Research Council Investigator Grant (GNT2009432). RP, JA ST, work for project partners, however no funders had a role in the conceptualization, design, data collection, analysis, decision to publish, or preparation of the manuscript.

## Ethics Statement

This study was approved by the Flinders Human Research Ethics Committee (HREC 7307).

## Conflicts of Interest

The paper was allocated to an independent Health Promotion Journal of Australia Associate Editor and be processed through the usual blind peer review process. Carmel Williams had no involvement in the review process once the paper was allocated to the Associate Editor.

## Supporting information


**File S1:** hpja70230‐sup‐0001‐Supinfo.docx.
**File S2:** hpja70230‐sup‐0001‐Supinfo.docx.
**File S3:** hpja70230‐sup‐0001‐Supinfo.docx.

## Data Availability

The data that support the findings of this study are available on request from the corresponding author. The data are not publicly available due to privacy or ethical restrictions.
