# Supplementary material for: Supporting Decision Making in Early Years Initiatives: Co‐Designing a Child Health Evidence Dashboard
Source: Health Promot J Austr. 2026 Aug 11;37(4):e70230. doi: 10.1002/hpja.70230 (PMC13461270; doi:10.1002/hpja.70230)
Supplement: Supplementary file 1 [file HPJA-37-0-s001.docx]

**Supplementary file 1**

**Table 1** GRIPP2 long form

| Section and topic | Item | Reported on page No |
| --- | --- | --- |
| Section 1: Abstract of paper | |  |
| 1a: Aim | Report the aim of the study | Y |
| 1b: Methods | Describe the methods used by which patients and the public were involved | Y |
| 1c: Results | Report the impacts and outcomes of PPI in the study | Y |
| 1d:Conclusions | Summarise the main conclusions of the study | Y |
| 1e: Keywords | Include PPI, “patient and public involvement,” or alternative terms as keywords | Next-users |
| Section 2: Background to paper | |  |
| 2a: Definition | Report the definition of PPI used in the study and how it links to comparable studies | Y |
| 2b: Theoretical underpinnings | Report the theoretical rationale and any theoretical influences relating to PPI in the study | Y |
| 2c: Concepts and theory development | Report any conceptual models or influences used in the study | Y- iKT / KTA |
| Section 3: Aims of paper | |  |
| 3: Aim | Report the aim of the study | Y |
| Section 4: Methods of paper | |  |
| 4a: Design | Provide a clear description of methods by which patients and the public were involved | Y |
| 4b: People involved | Provide a description of patients, carers, and the public involved with the PPI activity in the study | Y |
| 4c: Stages of involvement | Report on how PPI is used at different stages of the study | Y |
| 4d: Level or nature of involvement | Report the level or nature of PPI used at various stages of the study | Y – Methods and Discussion |
| Section 5: Capture or measurement of PPI impact | |  |
| 5a: Qualitative evidence of impact | If applicable, report the methods used to qualitatively explore the impact of PPI in the study | Y |
| 5b: Quantitative evidence of impact | If applicable, report the methods used to quantitatively measure or assess the impact of PPI | Y |
| 5c: Robustness of measure | If applicable, report the rigour of the method used to capture or measure the impact of PPI | Y |
| Section 6: Economic assessment | |  |
| 6: Economic assessment | If applicable, report the method used for an economic assessment of PPI | N/A |
| Section 7: Study results | |  |
| 7a: Outcomes of PPI | Report the results of PPI in the study, including both positive and negative outcomes | Y |
| 7b: Impacts of PPI | Report the positive and negative impacts that PPI has had on the research, the individuals involved (including patients and researchers), and wider impacts | Y - Discussion |
| 7c: Context of PPI | Report the influence of any contextual factors that enabled or hindered the process or impact of PPI | N/A |
| 7d: Process of PPI | Report the influence of any process factors, that enabled or hindered the impact of PPI | Y - Discussion |
| 7ei: Theory development | Report any conceptual or theoretical development in PPI that have emerged | N/A |
| 7eii: Theory development | Report evaluation of theoretical models, if any | N/A |
| 7f: Measurement | If applicable, report all aspects of instrument development and testing (eg, validity, reliability, feasibility, acceptability, responsiveness, interpretability, appropriateness, precision) | N/A |
| 7g: Economic assessment | Report any information on the costs or benefit of PPI | N/A |
| Section 8: Discussion and conclusions | |  |
| 8a: Outcomes | Comment on how PPI influenced the study overall. Describe positive and negative effects | Y |
| 8b: Impacts | Comment on the different impacts of PPI identified in this study and how they contribute to new knowledge | Y |
| 8c: Definition | Comment on the definition of PPI used (reported in the Background section) and whether or not you would suggest any changes | Y |
| 8d: Theoretical underpinnings | Comment on any way your study adds to the theoretical development of PPI | Y |
| 8e: Context | Comment on how context factors influenced PPI in the study | N/A |
| 8f: Process | Comment on how process factors influenced PPI in the study | Y- strengths and limitations |
| 8g: Measurement and capture of PPI impact | If applicable, comment on how well PPI impact was evaluated or measured in the study | N/A |
| 8h: Economic assessment | If applicable, discuss any aspects of the economic cost or benefit of PPI, particularly any suggestions for future economic modelling. | N/A |
| 8i: Reflections/critical perspective | Comment critically on the study, reflecting on the things that went well and those that did not, so that others can learn from this study | Y - strengths |

PPI=patient and public involvement

**Supplementary file 2**

**Table 1.** Summary of workshop methods

**Figure 1:** Miro whiteboard for activity of brainstorming and prioritising content

**Figure 2:** Miro whiteboard for activity of brainstorming features and functions
**Figure 3:** Miro whiteboard for activity of collecting feedback on the dashboard navigation flow diagram to inform prototype development (Workshop 3)

**Table 1.** Summary of workshop methods

| **Workshop number and topic** | **Workshop activities** | **Pre/post activities** |
| --- | --- | --- |
| 1. Uses and content | Demonstration:   - TOPCHILD evidence base and information included - Conceptual diagram + predetermined uses   Activities:   - Voting on dashboard uses and idea generation of additional uses - Break out rooms for idea generation of additional content (Figure 1) - Prioritise content (need to know, good to know, don’t need) | Pre-workshop:   - Pre-reading of the TOPCHILD evidence base   Post-workshop:   - Home-work survey to prioritise content ideas (need to know) |
| 2. Features and functionality | Demonstration:   - Example websites   Activities:   - Slido poll of likes and dislikes - Breakout rooms for idea generation using prompts (Figure 2) | Pre-workshop:   - Pre-reading of example websites and prompts   Post-workshop:   - Home-work survey to vote on ideas |
| 3. Design | Demonstration:   - Website navigation flow diagram - Demonstration of example websites   Activities:   - Group feedback of the navigation (Figure 3) - Group discussion of design likes/dislikes | Pre-workshop:   - Example websites and prompts/questions - Survey of design likes/dislikes |
| 4. Prototype feedback | Demonstration:   - Prototype   Activities:   - Individual exploration of the prototype - Slido feedback (like, wish, wonder) - Group discussion of feedback + live iterations of the prototype | Post-workshop:   - Survey of like, wish, wonder for additional prototype feedback |
| 5. Barriers and enablers | Activities:   - Group discussion of enablers, barriers and ideas for evaluation and implementation | Pre-workshop:   - Survey of enablers, barriers and implementation |

***Figure 1:*** *Miro whiteboard for activity of brainstorming and prioritising content
(Workshop 1, Group 2: Content subcategory ‘Resources and logistics to deliver the program’)*

***Figure 2:*** *Miro whiteboard for activity of brainstorming features and functions
 (Workshop 2, Group 2: Category ‘Features and functionality’)*

***Figure 3:*** *Miro whiteboard for activity of collecting feedback on the dashboard navigation flow diagram to inform prototype development (Workshop 3)*

**Supplementary file 3**

**Table 2:** Next-user requirements for a child health behaviour dashboard **Table 3:** Voting and prioritisation of content for the dashboard (post workshop 1)
**Table 4:** Voting and prioritisation of features and functions for the dashboard (post workshop 2)
**Table 5:** Participant feedback to improve the prototype (workshop 4)
**Text:** Evaluation and implementation

***Table 2:*** Next-user requirements for a child health behaviour dashboard

| ***Subcategory*** | ***Codes*** |
| --- | --- |
| Category: **Desired content for the dashboard** | |
| Who and what the initiative targets | Universal or priority populations  Stage of life course  Child health behaviours  Child development |
| Anticipated outcomes of the initiative | Child health behaviours Parent and family wellbeing  Initiative sustainability |
| Resources and logistics to deliver the initiative | How the initiative is delivered  Setting and organisation  Initiative cost  Equipment and technology needed  Resources and support for implementation  Resources for sustainability and scalability |
| Type of initiative and content/strategies | Type of initiative  Initiative background  Rationale  Materials provided to parents  Community led  Developed with consumer experience |
| Category: **Desired features and functions** | |
| Key features | Evidence hub of programs  Initiative/evidence summaries  Case studies  Tools and resources for implementation  Measurement tools for evaluation  ‘About us’ page  Website orientation video |
| Search and filtering | Ability to search Ability to filter |
| Navigation | Navigation tab across the top  Navigation by role |
| Outputs | Free and easy downloads  Evidence summaries Links to publications  Videos, factsheets and resources |
| Social sharing functions | Ability to share resources on social media  Ability to contact others who have delivered initiatives |
| Accessibility | Device compatibility |
| Category: **Desired design of the dashboard** | |
| Design features and user experience | Easy to navigate Simple design and layout  Intuitive to use |
| Aesthetic | Visually attractive  Use of white space  Relevant visuals and icons  Photography showing diversity |
| Readability | Big and clear font  Concise text  Text broken up with visuals  Headings and sub-headings  Clear and intuitive language |
| Content organisation and categorisation | Organisation by topic, setting or target audience  Date when the information was last updated  Matrix to link all relevant information to applied filter |

**Table 3**: Voting and prioritisation of content for the dashboard (Post workshop 1)

| **Information sources** | **Total tally (n=8)** **†** | **Within workshop prioritisation activity (separated into two groups)** |
| --- | --- | --- |
| **Who and what the program targets** | | |
| Stage of the life course targeted | 6 |  |
| Population targeted | 5 | Group 1 and 2 identified as must know |
| Child behaviours targeted | 5 | Group 1 and 2 identified as must know |
| Priority population targeted | 4 | Group 1 and 2 identified as must know |
| Child development targeted | 4 |  |
| Caregivers’ behaviours targeted | 3 |  |
| Family members targeted | 2 |  |
| **Resources and logistics to deliver the program** | | |
| How the program is delivered | 6 | Group 2 identified as must know |
| Program duration | 6 | Group 2 identified as must know |
| Program sustainability and scalability | 6 | Group 2 identified as must know |
| Setting and organisation where the program is delivered | 5 | Group 2 identified as must know |
| Equipment and technology required | 5 | Group 2 identified as must know |
| Training for facilitators | 5 | Group 2 identified as must know |
| Resources for program implementation | 5 | Group 1 and 2 identified as must know. |
| Strategies for embedding to routine service | 5 |  |
| Facilitator background | 4 |  |
| Funder of the program | 4 |  |
| Cost per participant | 3 |  |
| Cost of program | 2 |  |
| Expectations and enablers for participants | 2 |  |
| **Type of program and content/strategies** | | |
| Type of program delivery | 6 | Group 2 identified as must know |
| Materials provided to parents | 5 | Group 2 identified as must know |
| Community led | 5 | Group 1 identified as must know |
| Program rationale | 4 |  |
| Program background | 4 |  |
| Developed with consumer experience | 4 |  |
| Types of strategies for parents | 3 | Group 2 identified as must know |
| Interactive involvement from parents | 3 |  |
| Ability to tailor the program to individual parents | 2 |  |
| Theory based | 2 |  |
| **Anticipated outcomes of the program** | | |
| Impact on child health behaviours | 6 | Group 2 identified as must know |
| Impact on sustainability of the program | 5 |  |
| Impact of parent and family wellbeing | 5 |  |
| Impact on child development | 4 |  |
| Impact of program reach | 4 |  |
| Impact on food literacy | 4 |  |
| Impact on parenting | 3 | Group 2 identified as must know |
| Cost-benefit | 2 |  |
| Impact on food security | 2 |  |
| Impact on child weight status | 1 |  |

†n=2 started and did not finish the survey

**Table 4:** Voting and prioritisation of features and functions for the dashboard (Post workshop 2)

| **Features and functions ‡** | **Total tally (n=6)** | **†Project team prioritisation** |
| --- | --- | --- |
| Simple and intuitive | 6 | High |
| Tabs with simple headings and subheadings | 6 | High |
| Search filters | 6 |  |
| Section for tools and resources to support implementation and delivery | 5 | High |
| Evidence summaries/overviews | 5 | High |
| Free downloads e.g. publications, fact sheets, summaries, resources | 5 |  |
| Evidence hub of programs | 4 | High |
| Evidence rating systems (e.g. for cost, effectiveness) | 4 |  |
| Filtering functionality | 4 |  |
| Interactive map | 4 |  |
| Short video or text on the landing page | 3 |  |
| Website content page | 3 |  |
| Measurement tools and instruments for evaluation | 3 | High |
| Ability to contact others who have delivered programs | 3 |  |
| Options for the output format e.g. videos, factsheets, podcasts | 2 |  |
| Device compatibility | 2 | High |
| Page description | 1 |  |
| Decision tree to support process of finding evidence | 0 |  |
| **Evidence filters** |  |  |
| Topic e.g. nutrition, physical activity | 6 | - |
| Cost | 6 | - |
| Population | 5 | - |
| Location | 5 | - |
| Outcomes | 5 | - |
| Duration | 4 | - |
| Setting | 3 | - |

†project team priorities based on conceptual planning/broader project planning

‡ Development of the dashboard was iterative so there are features/functions that came up in other workshops/reinforced in discussions/feedback sessions that inform the category in the manuscript.

**Table 5:** Participant feedback to improve the prototype (Workshop 4)

| ***Page*** | ***Participant feedback*** |
| --- | --- |
| ***Home page*** | - Move the orientation video upfront in the hero banner - Move link to resources upfront in the first section - Add ‘evidence summaries’ under ‘type of resources’ - Add call to action (the link to resources) on the first section of the home page - Smaller hero banner to see what’s below - Too much scrolling |
| ***Resources page*** | - Add a filtering slider for date range - Add a filtering slider for program duration - Colour code resources according to their type (e.g. case study, evidence summary, program summary, measurement tool) - Add ability to browse by category/topic or resource type - Add resources in list form - Add in date of resources |
| ***About us*** | - Move the team (e.g. the team involved in developing the website) in ‘about us’ to a different page - Shorter ‘about us’ page |
| ***FAQ page*** | - Add the FAQ as its own page - Consider adding content for program FAQ and dashboard related FAQ |
| ***Other*** | - Add in colour - Make it clearer about the ability to log in, bookmark and share |

***Evaluation and implementation***

Participants brainstormed ideas for evaluating and implementing the dashboard, providing considerations for the context of timelines required to meaningfully use the dashboard. Ideas for evaluating the dashboard were involving collaborators and stakeholders to use the dashboard, use of dashboard tracking metrics such as number of visits or average time spent on the dashboard, feedback questionnaires and focus groups. Suggestions for implementation included stakeholder mapping for potential users, soft launching in a pilot phase, promotion through project partners and social media with ongoing communication and engagement strategies.

***Barriers to use of the dashboard***

The barriers that may impact use of the dashboard identified subcategories for lack of relevance, access barriers, user experience barriers and design and readability barriers.

Barriers impacting access to the dashboard included potential firewall restrictions within organisations “*firewall issues with my organisation*” (Anonymous, Pre-workshop 5 survey), additionally recognising cost or being incompatible with different devices. Participants described the lack of relevance being a barrier with considerations for limited high-quality or up to date information or lack of resources available limiting the usability, “*If the dashboard does not provide enough practical/usable information about interventions/research to enable users to analytically consider the info.”* (Anonymous, Pre-workshop 5 survey). In addition, forgetting its existence would prevent their use. They identified barriers related to user experience with examples of the dashboard being difficult to navigate or too complicated, highlighting that requiring training to use it will inhibit use “*I don’t want to go to a work a class for an hour to learn how to use something I haven't got the time so*” (Participant 9, Workshop 1). They described use of excessive jargon, scrolling and clicks will impact their user experience. Further, barriers related to design and readability that can deter use included too much text and data, not attractive, use of distracting features or too busy on the screen.
